# Clinicopathologic features and outcomes following surgery for pancreatic adenosquamous carcinoma

**DOI:** 10.1186/1477-7819-6-95

**Published:** 2008-09-03

**Authors:** Jun-Te Hsu, Han-Ming Chen, Ren-Chin Wu, Chun-Nan Yeh, Ta-Sen Yeh, Tsann-Long Hwang, Yi-Yin Jan, Miin-Fu Chen

**Affiliations:** 1 Department of Surgery, Chang Gung Memorial Hospital, Chang Gung University College of Medicine, Taoyuan, Taiwan; 2 Department of Surgery, Chung Shan Medical University Hospital, Chung Shan Medical University, Taichung, Taiwan; 3 Department of Pathology, Chang Gung Memorial Hospital, Chang Gung University College of Medicine, Taoyuan, Taiwan

## Abstract

**Background:**

Pancreatic adenosquamous carcinoma (ASC) is a rare pancreatic malignancy subtype. We investigated the clinicopathological features and outcome of pancreatic ASC patients after surgery.

**Methods:**

The medical records of 12 patients with pancreatic ASC undergoing surgical treatment (1993 to 2006) were retrospectively reviewed. Survival data of patients with stage IIB pancreatic adenocarcinoma and ASC undergoing surgical resection were compared.

**Results:**

Symptoms included abdominal pain (91.7%), body weight loss (83.3%), anorexia (41.7%) and jaundice (25.0%). Tumors were located at pancreatic head in 5 (41.7%) patients, tail in 5 (41.7%), and body in 4 (33.3%). Median tumor size was 6.3 cm. Surgical resection was performed on 7 patients, bypass surgery on 3, and exploratory laparotomy with biopsy on 2. No surgical mortality was identified. Seven (58.3%) and 11 (91.7%) patients died within 6 and 12 months of operation, respectively. Median survival of 12 patients was 4.41 months. Seven patients receiving surgical resection had median survival of 6.51 months. Patients with stage IIB pancreatic ASC had shorter median survival compared to those with adenocarcinoma.

**Conclusion:**

Aggressive surgical management does not appear effective in treating pancreatic ASC patients. Strategies involving non-surgical treatment such as chemotherapy, radiotherapy or target agents should be tested.

## Background

Adenocarcinoma accounts for the majority of pancreatic malignancies. Adenosquamous carcinoma (ASC) of the pancreas is an unusual variant of pancreatic neoplasm [1-4], and is characteristic by histological patterns of both ductal adenocarcinoma and squamous carcinoma within the same tumor. The prognosis of this rare tumor appears to be even less favorable than the common invasive ductal tumor with few patients surviving more than 1 year after surgical resection [4]. Most of studies on this disease have been small series or single case reports, and few studies have investigated the clinicopathologic features and outcome of patients with pancreatic ASC following surgical treatment [1,2,5,6]. Therefore, medical records of 12 patients with pancreatic ASC treated surgically at Chang Gung Memorial Hospital (CGMH), Taoyuan in the past 14 years were retrospectively reviewed.

## Methods

A total of 637 patients with pancreatic malignancies underwent surgical treatment at CGMH between January 1993 and December 2006. Adenocarcinoma was diagnosed in 530 patients and ASC in 12. Institutional Review Board approval was obtained and medical records of 12 patients with pancreatic ASC were retrospectively reviewed. Preoperative imaging studies employed abdominal ultrasonography, abdominal computed tomography (CT)/magnetic resonance imaging (MRI), and endoscopic retrograde cholangiopancreatography (ERCP). Serum tumor markers such as carcinoembryonic antigen (CEA) and carbohydrate antigen 19-9 (CA 19-9) were measured preoperatively. One patient had a preoperative fine needle tumor biopsy. Intraoperative radiotherapy and postoperative chemotherapy were performed in 2 patients and 7 patients, respectively. Tumor stage and TMN stage were defined according to the sixth edition of American Joint Committee on Cancer for pancreatic carcinoma [7] based on the histopathologic examination of surgical specimens and clinical findings such as imaging studies and intraoperative records. To further elucidate the outcome following surgical resection for pancreatic ASC from more common pancreatic adenocarcinoma, patients with stage IIB pancreatic adenocarcinoma undergoing surgical resection were also extracted from our databank (at the same studying period as pancreatic ASC). Survival data of patients with stage IIB pancreatic adenocarcinoma and ASC undergoing surgical resection were compared. Survival rate was calculated and graphs plotted using Kaplan-Meier method. Differences in survival curves between the groups were compared by the log-rank test. A *p*-value less than 0.05 were defined as statistically significant. All statistical analyses were performed with SPSS for Windows, version 11.5 (Statistical Package for the Social Science, SPSS, Inc., Chicago, Illinois).

## Results

The demographic features of 12 patients with pancreatic ASC including 5 men and 7 women (age range, 32 to 79 years; median, 71 years) are shown in Table 1. Symptoms were abdominal pain in 11 patients (91.7%), body weight loss in 10 (83.3%), anorexia in 5 (41.7%), jaundice in 3 (25.0%). Ten patients had 13 comorbidities including hypertension in 5, diabetes mellitus in 4 and peptic ulcer in 3, and heart disease in 1. Laboratory studies revealed anemia in 9 patients (75.0%), elevated total bilirubin levels in 3 (25.0%), and elevated alkaline phosphatase levels in 3 (25.0%). Elevated serum CEA levels and CA 19-9 levels were identified in 10 patients (83.3%), respectively. Three patients underwent ERCP, which identified tumor obstruction of the pancreatic head duct. All patients underwent abdominal CT or MRI, which accurately determined and localized a pancreatic tumor.

**Table 1:** Demographics of 12 patients with pancreatic adenosquamous carcinoma.

Case	Age/sex	Symptoms	Comorbidity	Laboratory data			Tumor markers	
				
				Hb (g/dL)	Bil (T) (mg/dL)	Alk-P (U/L)	CEA (ng/mL)	CA 19-9 (U/mL)
1	73/F	Abd pain, BWL, jaundice	HTN	10.4	15.7	258	262	240
2	66/M	BWL, jaundice, anorexia	DM	10.2	14.8	438	13.1	>240
3	65/F	Abd pain, BWL, diarrhea	HTN, heart disease	13.4	0.4	89	25.2	15.7
4	63/M	Abd pain, BWL	Peptic ulcer	13.2	0.5	92	10.2	135
5	78/M	Abd pain, anorexia	Nil	10.6	0.7	78	12.4	142.4
6	79/M	Abd pain, BWL, anorexia, abd mass	Nil	12.6	0.5	95	17.6	8.5
7	38/F	Abd pain, BWL, jaundice	DM	10.7	23.2	192	14.5	138
8	79/F	Abd pain, dizziness, malaise	HTN, DM, Peptic ulcer	7.0	0.8	59	2300	>240
9	76/F	Abd pain, BWL	HTN	11.3	0.5	93	5.34	129
10	32/M	Abd pain, BWL	Peptic ulcer	15.2	0.5	84	1.79	84
11	69/F	Abd pain, BWL, anorexia	DM	9.4	0.5	66	83.74	>240
12	78/F	Abd pain, BWL, anorexia, malaise	HTN	10.6	0.3	68	0.57	160.9

Abd, abdominal; Alk-P, alkaline phosphatase; Bil (T): total bilirubin; BWL, body weight loss; CA19-9, carbohydrate antigen 19-9 (< 37 U/mL); CEA, carcinoembryonic antigen (< 5 ng/mL); DM, diabetes mellitus; Hb, hemoglobin; HTN, hypertension.

**Figure 1:**
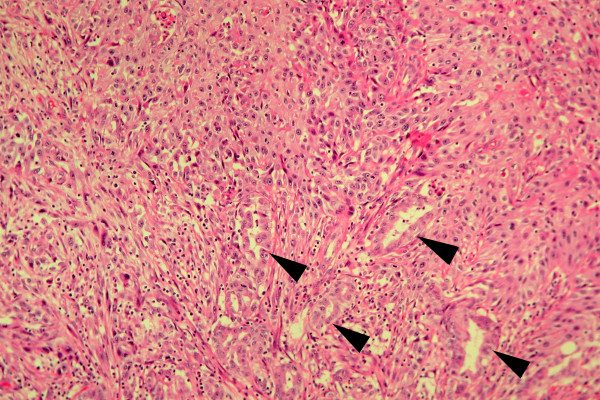
Histopathology in a patient with pancreatic tumor shows glandular adenocarcinoma foci (black arrowheads) and nests of squamous cell carcinoma (upper middle part), consistent with adenosquamous carcinoma (Hematoxylin-Eosin stain, original magnification ×100).

Table 2 demonstrates the details of tumor characteristics, management and prognosis of 12 patients with pancreatic ASC. The tumors were located at pancreatic head in 5 (41.7%) patients, tail in 5 (41.7%), and body in 4 (33.3%). Tumor size ranged from 3.5 to 8 cm with a median of 6.3 cm. The lesions from the resected specimens were firm with light tan to yellowish colors and had merged imperceptibly with the surrounding pancreatic parenchyma. Histologically, tumors were a mixture component of adenocarcinoma and squamous cell carcinoma (Figure 1). The rates of squamous component in the tumor tissue ranged from 40 to 90% in patients undergoing surgical resection. Lymph node metastases were identified in 11 patients (91.7%). Encasement of superior mesenteric artery by the tumor was found during operation in 5 patients, and carcinomatosis in 1 patient. Surgical resection including pancreaticoduodenectomy (PD) and subtotal or distal pacreatectomy along with total gastrectomy or splenectomy was performed in 7 patients. R0 (radical) resection was identified in 5 patients and R1 resection in 2 (cases 9 and 11). Five patients underwent laparotomy followed by intra-operative biopsy of the pancreatic tumor and three received bypass surgery. Intraoperative irradiation and postoperative chemotherapy were carried out in 2 and 7 patients, respectively. Tumor stage was IIB in 7 patients, III in 4 and IV in 1.

**Table 2:** Details of tumor characteristics, management, and prognosis of 12 patients with pancreatic adenosquamous carcinoma.

Case	Tumor location	Size (cm)	Operative method	Intraoperative irradiation	Postoperative chemotherapy	Stage* (TNM)	Survival (months)
1	Head	6	Biopsy, bypass	ND	ND	III (T4N1M0)	4.04
2	Head	3.5	PD	ND	ND	IIB (T2N1M0)	2.50
3	Body and tail	8	Biopsy, bypass	ND	ND	IV (T4N1M1)	1.12
4	Head	6	Biospy	1,800 cGy	ND	III (T4N0M0)	22.42
5	Tail	8	Biopsy	2,000 cGy	Gemcitabine	III (T4N1M0)	5.42
6	Body	8	Biopsy, bypass	ND	Tegafur	III (T4N1M0)	4.41
7	Head	3.8	PD	ND	Gemcitabine, Fluorouracil	IIB (T2N1M0)	6.84
8	Head	5.5	PD	ND	Gemcitabine	IIB (T3N1M0)	6.51
9	Body	7	subtotal P, total G, S	ND	Tegafur, Uracil	IIB (T3N1M0)	11.84
10	Tail	5	distal P, S	ND	ND	IIB (T2N1M0)	10.82
11	Tail	8	distal P, total G, S	ND	Gemcitabine, Cisplatin	IIB (T3N1M0)	3.68
12	Body and Tail	6.5	subtotal P, S	ND	Gemcitabine	IIB (T3N1M0)	4.08

G, gastrectomy; ND, not done; P, pancreatectomy; PD, pancreaticoduodenectomy; S, splenectomy; *, clinical and pathological.

**Figure 2:**
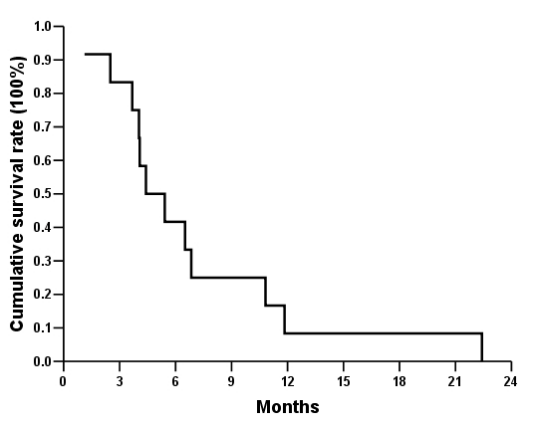
Cumulative survival rates of 12 patients with pancreatic adenosquamous carcinoma after surgery.

**Figure 3:**
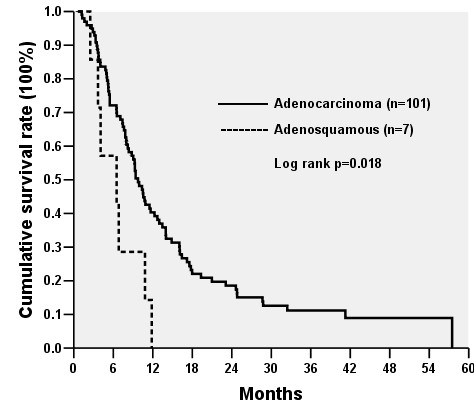
Cumulative survival rates of patients with stage IIB pancreatic adenocarcinoma and adenosquamous carcinoma undergoing surgical resection.

There was no surgical mortality. The time of follow-up ranged from 0.79 to 122.66 months with a median of 6.49 months. Figure 2 shows the cumulative survival rates of 12 patients with pancreatic ASC with a median of 4.41 months, ranging from 1.12 to 22.42 months. Eleven of 12 patients with pancreatic ASC died in one year after surgery with one-year survival rate of 8.3% (95% confidence interval, 0.0–24). Figure 3 demonstrates cumulative survival rates of stage IIB pancreatic adenocarcinoma (n = 101) and ASC (n = 7) patients undergoing surgical resection. Patients with pancreatic ASC had shorter median survival compared to those with adenocarcinoma (6.51 months vs. 9.76 months, *p *= 0.018).

## Discussion

The first report of ASC is credited to Herxheimer in 1907 [8]. This admixed tumor has been seen more commonly in other organ systems where adenocarcinomas are generally found, such as the stomach [9], intestine [10] and uterus [11]. It has also been identified in the esophagus [12], anus [13] and vagina [14] where squamous cell carcinomas predominate. In the present studies, the incidence of pancreatic ASC was 1.9% (12/637), within the range of 0.9 to 3.8% reported in the literatures [2-4]. The histogenesis of pancreatic ASC remains unclear. There are numerous possibilities that account for the presence of a squamous element where adenocarcinoma is expected. Four theories regarding the histogenesis of adenosquamous carcinoma may be summarized as follows: adenocarcinoma transforming into squamous cell carcinoma; bipotential undifferentiated cell origin; collision tumor; and squamous metaplasia origin [5].

Madura et al. [1] reported that most patients with pancreatic ASC are males in their 60s and frequently located at the head of the pancreas. Different from their findings, more females were identified in our patients, and the patient median age was 71 years. Moreover, our results show that the tumor location was evenly distributed at the pancreatic head, body, or tail. Symptoms of our patients with pancreatic ASC were abdominal pain (92%), body weight loss (83%), anorexia (42%) and jaundice (25%) similar to those of pancreatic adenocarcinoma [15].

Accurate preoperative diagnosis of pancreatic ASC is made with great difficulty since there are no investigations of its defining characteristics in imaging studies that would differentiate it from the more common pancreatic exocrine neoplasm [1]. Nevertheless, studies have indicated that cytological examination of pure pancreatic juice obtained by endoscopic retrograde pancreatic juice aspiration is a useful modality for the preoperative diagnosis [16]. Rahemtullah et al. [17] also reported that cytological features derived from fine-aspiration biopsy are diagnostic of pancreatic ASC. Furthermore, imaging studies by Nabae et al. [15] showed that the presence of central necrosis in a huge infiltrative pancreatic tumor is suggestive of the diagnosis of ASC. Moreover, a tumor might selectively take up gallium 67 and be visualized by nuclear scanning which is useful in detecting this rare pancreatic tumor [18]. In the present studies, no patient had central necrosis at the pancreatic tumor on abdominal imaging studies indicating a diagnosis of pancreatic ASC. Besides, the preoperative fine needle biopsy of the tumor was performed in 1 patient, which revealed adenocarcinoma.

As shown in table 2, 11 patients with pancreatic ASC (92%) died within 12 months despite aggressive surgical management along with intraoperative irradiation or postoperative chemotherapy. The median cumulative survival of 12 patients was 4.92 months (Figure 2). Furthermore, median survival of 7 patients undergoing surgical resection was 6.51 months. These results were similar to that obtained by Madura et al. [1], who reported that 72 patients survived with an average age of 5.7 months, regardless of whether or not surgical resection was performed. To our surprise, 1 patient in our series who had no lymph node involvement without undergoing surgical resection and received intraoperative irradiation had a survival of 22.42 months. No lymph node metastasis and the potential benefit of intraoperative radiation therapy might explain his long survival.

Once pancreatic ASC is identified either preoperatively or intraoperatively, the choice of treatment becomes a complex decision as survival is typically dismal [1]. In this regard, although PD has been shown to be performed with a very low mortality rate (<4%) in specialized high-volume centers, the incidence of postoperative morbidity can be as high as 30% to 40% [19,20]. Furthermore, a significant high mortality rate (25%) has been reported in the patient subgroup with significant preoperative comorbidities [20]. Thus, anesthesia risks and complications following major surgery in pancreatic ASC patients along with severe medical diseases should be considered before operation. Moreover, we observed that median survival of patients (stage IIB) with pancreatic ASC undergoing surgical resection was 6.51 months, significantly shorter (*p *= 0.018) than patients with stage IIB pancreatic adenocarcinoma receiving resection (median survival, 9.76 months; Figure 3), suggesting more aggressive biology of pancreatic ASC than adenocarcinoma. Moreover, nodal metastases were identified in 92% (11/12) of our patients, which might reflect the disease entity tending to have lymph node involvement and at least partly explained the poor prognosis of this virulent tumor.

It should be noted that this study was based on a retrospective review of patients undergoing surgery. Pancreatic malignancy patients who were not diagnosed as pancreatic ASC without tissue proof treated non-surgically were not enrolled in this study. Whether surgical resection or non-surgical management such as chemotherapy, radiotherapy, chemo-radiotherapy or target therapy would provide survival benefits to patients with pancreatic ASC remains unknown. More studies are necessary to confirm this.

## Conclusion

Pancreatic ASC is a rare pancreatic neoplasm subtype. Abdominal pain and body weight loss are the two predominant symptoms. Distribution of ASC is even in the pancreas, and the tumor size is big at the time of diagnosis. Pancreatic ASC tends to have nodal metastases and has a dismal outcome despite surgical resection. In this limited case study, aggressive surgical management does not appear effective in treating pancreatic ASC patients. Strategies involving non-surgical treatment such as chemotherapy, radiotherapy or target agents should be tested.

## Competing interests

The authors declare that they have no competing interests.

## Authors' contributions

HJT: planning, study design, data collection and analysis, drafting, and revising the manuscript. CHM: planning, study design and analysis, surgical management of patients. WRC: pathological review of surgical specimens, preparing the pathological figure. YCN: study design and analysis, surgical management of patients, YTS: study design and analysis, surgical management of patients. HTL: study design and analysis, surgical management of patients, revising the manuscript. JYY: study design and analysis, surgical management of patients. CMF: study design and analysis, surgical management of patients. All authors read and approved final manuscript.
